# Individualized 3D Planning for Hip Reconstruction in Cerebral Palsy: Study Protocol

**DOI:** 10.3390/jcm15072636

**Published:** 2026-03-30

**Authors:** Britta K. Krautwurst, Thomas Dreher, Franziska L. Hatt, Bastian Sigrist, Tobias Götschi, Domenic Grisch

**Affiliations:** 1 Department Pediatric Orthopedics and Traumatology, University Children’s Hospital Zurich, 8008 Zurich, Switzerland; 2 Department of Pediatric Orthopedics, Balgrist University Hospital Zurich, University of Zurich, 8008 Zurich, Switzerland; 3 Research in Orthopedic Computer Science (ROCS), University Hospital Balgrist, University of Zurich, 8008 Zurich, Switzerland; 4 Clinical Trials Unit, University Hospital Balgrist, University of Zurich, 8008 Zurich, Switzerland

**Keywords:** study protocol, cerebral palsy, hip dysplasia, acetabular morphology, CAOS guided surgery

## Abstract

**Background:** In children with cerebral palsy, bony acetabular deficiencies are common and may be associated with progressive hip subluxation, abnormal joint loading, and ultimately hip dislocation. Hip reconstruction surgery is typically performed to prevent dislocation, and this includes acetabular reshaping using acetabuloplasty. The location of acetabular deficiency may vary among individuals; however, only radiographs are used for planning and intraoperative correction in many centers. Precise reconstruction and preop planning are necessary for the accurate correction of acetabular coverage. This study compares conventional hip reconstruction with a 3D-guided technique using individual preop 3D planning and 3D-printed guides during surgery to determine which method allows for a more accurate correction. We hypothesize that the patient-specific 3D planning leads to more precise anatomical correction of acetabular coverage compared to conventional freehand osteotomy. **Methods**: This study was registered in the German Clinical Trial Register (DRKS-ID: DRKS00031356) on 14 July 2023. In a randomized controlled trial, various imaging-based parameters were used to assess the bony anatomy preoperatively and postoperatively. Preoperative and 6-week postoperative computed tomography (CT) scans are part of routine clinical care. Additionally, an immediate postoperative CT scan was performed. One hip was operated on using individualized 3D preoperative planning, while the other hip was corrected using a conventional surgical approach. A standardized subtrochanteric osteotomy was performed for the varisation, derotation, and shortening of the proximal femur. This osteotomy was followed by acetabuloplasty under fluoroscopic control. For the 3D-planned operation, patient-specific cutting and repositioning guides were produced based on preoperative CT imaging. Patients with bilateral cerebral palsy (GMFCS levels I–V), aged 4–18 years, with an open triradiate growth plate and a migration index ≥ 40% in at least one hip were included. In a preliminary retrospective part, this project reproduces the existing three-dimensional acetabular index (3-DAI) and compares it with established radiographic methods to determine the utility and reliability of a reconstructed 3D CT measurement technique. A further component of the retrospective part is the creation of an age-adjusted database of typically developed hips and the development of a 3D head coverage index (3D-HCI) as a new 3D parameter to express acetabular coverage; therefore, it will be used as a secondary parameter and correlated to the 3DAI in the prospective part. **Conclusions:** Improved precision may have meaningful clinical implications for long-term joint congruency, load distribution, pain, and mobility outcomes.

## 1. Introduction

### 1.1. Background

In the first years of life, children typically show a steep acetabulum and increased femoral anteversion (FAV) and caput collum diaphyseal angle (CCD), which normally decreases during growth [1]. This process is restricted in children with cerebral palsy (CP) depending on the ability to walk, which leads to hip dysplasia, hip subluxation, and ultimately dislocation in an average of 35% of patients, especially in patients who are unable to walk [2,3]. Ambulatory ability is graded by the Gross Motor Function Classification System (GMFCS) [4]. The risk for continued displacement is highest in GMFCS level V, with approximately 90%, and in GMFCS level IV, with 70% [5,6]. Causes are muscular imbalance associated with disturbed bone development because of lack of weight bearing. 

A dislocated hip may provoke pain and lead to further loss of function and often to limitations in important basic functions, such as lying, care, sitting, standing, and transfer [7,8]. For this reason, early hip reconstruction, considering all the deformities, is indicated to prevent loss of function. In the literature, recurrence rates of hip dislocation in children with CP were reported up to 23% despite the successful early radiological results after reconstruction [9,10,11,12,13,14,15]. A retrospective analysis of pediatric and adult patients with cerebral palsy undergoing surgery describes the risk of perioperative morbidity with 63% and mortality with 0.1% [16]. This highlights the importance of precise operation planning and implementation.

In addition to the clinical examination, the hip in CP is monitored using anteroposterior pelvic radiography. The migration index according to Reimers (MI) [17] describes the lateral femoral head migration with excellent validity [8]. The acetabular index (AI) shows the steepness of the acetabulum in the frontal plane and thus the degree of acetabular dysplasia. The validity is moderate, but the inter-rater reliability was found to be excellent [18]. The measurement accuracy using radiographs depends on the pelvic alignment, which is usually very inconsistent depending on patient compliance, muscle contractures, hypertonia, and spinal curvatures [19].

All these values only give a single-dimensional impression of the bony situation without considering the 3D morphology of the acetabular deficiency and the direction of the dislocation of the femoral head. Thus, in some centers, computed tomography (CT) with a three-dimensional reconstruction (3D-CT) is performed prior to reconstructive surgery. However, a well-defined, standardized measurement or planning method and normative data have not yet been established. The benefit of such additional imaging on treatment planning has not yet been examined.

Buckley et al. [20] described the morphological measurements of the acetabulum in CP in the transverse plane of a two-dimensional CT (2D-CT) using the anterior acetabular index, posterior acetabular index, axial acetabular index, and acetabular anteversion. Normal development of the acetabulum was described by Weiner et al. [21] using the axial acetabular index and the acetabular anteversion. The reliability of describing the acetabular shape in the transverse plane and the validity for the acetabular coverage in the preoperative status has been confirmed by Park et al. [22] but the concurrent validity for acetabular coverage after an osteotomy and the construct validity for osteotomy have been described as weak. Peterson et al. [23] showed the topography and orientation of the developing acetabulum in typically developing children using 3D-CT. The data suggest that the final region of the acetabulum to develop is the posterior region. 

**Figure 1:**
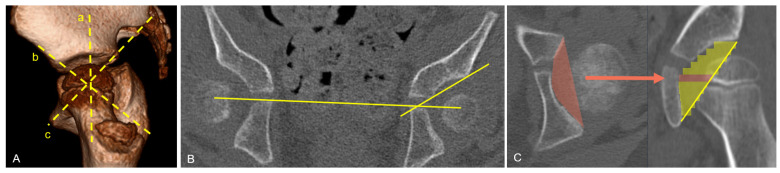
(**A**) Measurement of the superolateral index (a), anterosuperior index (b), and posterosuperior index (c). (**B**) Reformatted CT scan showing the anterosuperior index. (**C**) Calculation of the acetabular volume considering the slice thickness by integrating the area of the acetabulum [25].

Different 3D-CT measurement methods to analyze acetabular dysplasia in CP have been published. Kim et al. [24] described the locations of acetabular deficiency as posterior (37%), anterior [29%], midsuperior (15%), and mixed (19%). Quantitative measurements from Soo using axial CT suggested that the acetabular defect is also posterior [6]. Gose et al. described quantitatively the lateral opening angle, the sagittal inclination angle, and the CT migration percentage and showed a correlation to the acetabular index and a strong correlation to the migration index on an anteroposterior radiograph [18]. To measure acetabular defects more accurately, Chung et al. used the multiplanar reformation technique to determine the three-directional acetabular index (3-DAI) and the acetabular volume (AV) [25]. Figure 1 represents the three reformatted planes for calculating these variables. The anterosuperior index was determined in a plane defined by the anterior inferior iliac spine and the posterior aspect of the ischial tuberosity. The superolateral index was measured in a plane passing through the center of the acetabulum and oriented parallel to the reference line connecting the anterior superior ilia spine and the pubic symphysis. The posterosuperior index was assessed in a plane defined by the line connecting the pubic symphysis and the posterior inferior iliac spine. 

For a complete assessment of the acetabular coverage, the inclusion of the cartilage situation in a 3D manner would be desirable. Magnetic resonance imaging (MRI) offers the advantage of showing the cartilage condition and can also be performed using a three-dimensional reconstruction (3D-MRI), which is comparable [2,26,27] with the 3D-CT. Arthrography is a well-established method to make the cartilaginous parts of the joint congruence and the true deformity of the femoral head intraoperatively visible in one plane (anterior posterior) [28,29]. The limitations of both methods lie in the necessary anesthesia in the patient population with CP. Therefore, MRI is not yet used as a clinical standard and arthrography is used exclusively intraoperatively.

Within the computer-assisted orthopedic surgery (CAOS) research field, the combination of 3D preoperative planning and patient-specific navigation guides has been proven as an effective technique to improve precision for deformity and malunion corrections [30,31,32]. Furthermore, several methods have been proposed for three-dimensionally assessing the morphological and orthopedic parameters of femur and acetabulum [33,34,35]. Larson et al. and Liu et al. proposed a 3D method for calculating morphological parameters by projecting points of the acetabular rim on a sphere representing the femoral head [34,35]. However, these methods do not account for the real anatomical shape of the femoral head.

Even if 3D-CT was used, most of the methods proposed for pediatric hip surgery still rely on manual measurements on reformatted CT slices [36]. The work of Chung et al., 2006, describes the 3-DAI and AV, and in the follow-up work in 2008, the group demonstrates that the application is capable of assessing Dega pelvic osteotomies in CP [25,37]. However, the state of the art in CAOS in particular lacks validated methods for preoperatively simulating pediatric hip surgeries in a step-by-step fashion up to the postoperative outcome. Furthermore, only a few studies addressed the challenge of the surgical navigation of pediatric surgeries by means of patient-specific guides, and none of these evaluated efficiency for hip osteotomies [38,39,40].

### 1.2. Objectives

Primary research hypothesis (prospective part):

1. The improvement of the acetabular coverage is larger considering 3D data and the 3D-based correction with patient-specific guides compared to conventional surgical correction.

Null hypothesis: There is no improvement of the acetabular coverage considering 3D data and the 3D-based correction with patient-specific guides compared to conventional surgical correction.

Alternative hypothesis: The improvement of the acetabular coverage is larger considering 3D data and the 3D-based correction with patient-specific guides compared to conventional surgical correction.

Secondary research objectives (retrospective and prospective parts):

2. Creating an age-matched database from a typically developing reference group to describe the acetabular deficits and dislocation directions in CP measuring the head-shaft-angle (HSA), center-edge angle (CEA), CCD, AI, MI, 3-DAI, and AV (retrospective part).
3. Developing an algorithm describing the acetabular coverage and defining a new 3D-HCI (percentage) as an outcome parameter and correlating it with the MI, AI, 3-DAI, and AV (retrospective part).
4. Analysis if the 3D-HCI method is comparable, so as to assess the 3-DAI (prospective part).
5. Analysis if the 3D-HCI identifies hips with residual insufficient 3D coverage, which show normal values in the 3-DAI (prospective part).

## 2. Materials and Methods

This study was registered in the German Clinical Trial Register (DRKS-ID: DRKS00031356) on 14 July 2023.

### 2.1. Study Design and Setting

This study is a randomized clinical trial. It is monocentric and national.

To minimize a possible performance bias, double blinding is introduced. The selection bias is controlled by block randomization.

**Figure 2:**
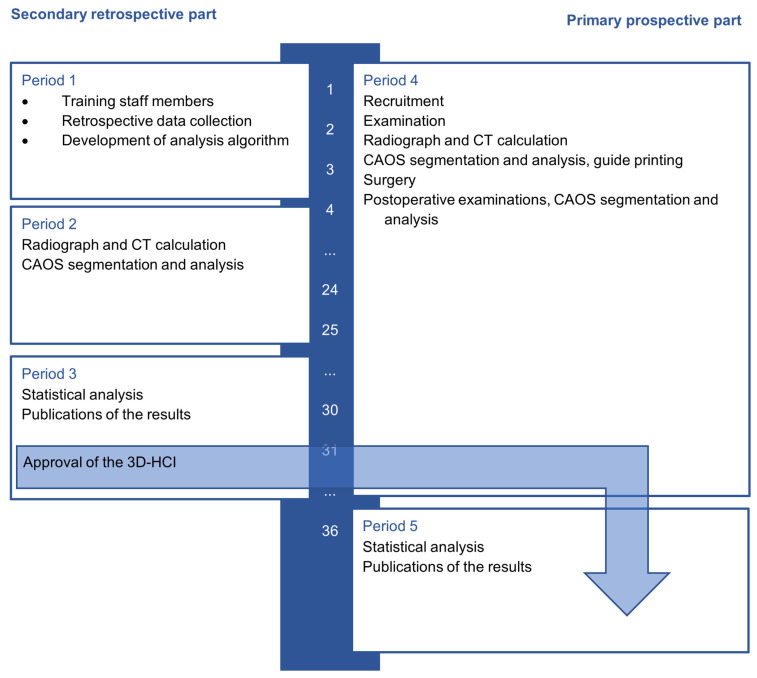
Timeline of the prospective and retrospective studies. The periods including their milestones are shown.

This study is structured in two parts, which run parallel to each other. The prospective part is a health-related intervention, categorized as another clinical trial category B. The second part is retrospective using existing data to create the new 3D head coverage index (3D-HCI) parameter (Figure 2).

#### Retrospective Part

The retrospective part includes two groups: one group of typically developing children with typically developed hips and hips of children of the same age with CP who have received a hip reconstruction. 

Children who received a pelvic CT due to another reason than hip dysplasia at the Children’s Hospital Zurich since the beginning of the electronic archiving of the radiological investigations in 2006 were screened for potential inclusion by an independent physician. 

An age-matched database with standard values of a typically developing reference group from 4 years until the closure of the triradiate cartilage were derived. The necessary information was obtained from the 3D-CT of typically developing hips. The values collected include AI, CEA, CCD, HSA, MI, 3-DAI, and AV.

An algorithm was developed to describe the acetabular coverage three-dimensionally and to compare the reference data with children with CP. This enabled us to describe the acetabular deficit and dislocation direction and represented the basis to develop a 3D-HCI. The index is part of the evaluation of the prospective study.

The project was planned for 36 months and divided into two studies. The secondary retrospective study took 30 months and is divided into one to three periods. The primary prospective study began at the same time as period 1 and took 36 months, divided into four to five periods.

### 2.2. Eligibility Criteria

Inclusion criteria:

– Data available digitally.
– CT with possible 3D reconstruction.
– Unilateral or bilateral cerebral palsy, GMFCS levels I to V (CP group).
– Age between 4 and 18 years.
– Open triradiate cartilage.
– No other neurological disease and at least one typically developing hip (reference group).
– Signed ethical consent.

Exclusion criteria:

– Incomplete data.
– Neuromuscular secondary diagnosis.

### 2.3. Outcome

Research question 2: In the retrospective part, an age-matched typically developing reference group was generated. The aim was to detect the acetabular deficit and dislocation direction in children with CP. For this purpose, different parameters (HSA, CEA, CCD, AI, MI, 3-DAI, and AV) were used. These are described descriptively. The values of the typically developing group are compared with those of the CP group.

**Figure 3:**
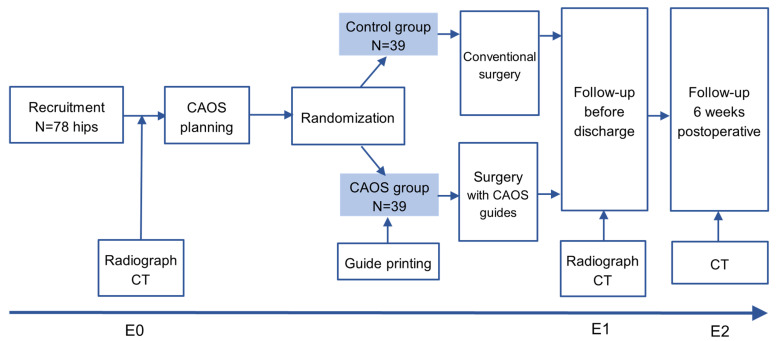
Timeline. Preoperative low-dose CT analysis (E0), analysis before discharge (E1), and 6 weeks postoperatively (E2).

Research question 3: As the main purpose of the retrospective part, an algorithm was developed to describe the acetabular coverage and dislocation directions three-dimensionally as a basis for the development of a 3D head coverage index (3D-HCI) relevant for outcome measurement. To evaluate this new index, it was correlated with the already existing parameters MI, AI, 3-DAI, and AV. The correlation was analyzed separately in both groups. The aim is to use the 3D-HCI as a new 3D parameter to express the acetabular coverage, and therefore, it will be taken as a secondary parameter in the prospective study (Figure 3).

#### Prospective Part

In a randomized controlled trial, 78 hips in 39 children with bilateral CP scheduled for a bilateral hip reconstruction surgery were assigned with one hip assigned to the CAOS group and the other hip to the control group. The indication for surgery was made during regular consultation at the Children’s University Hospital Zurich. The final consent to participate in the study was given in the following consultation, which always takes place in the clinical routine, in order to carry out the CT to plan the operation and to clarify any remaining questions.

All patients undergo a standardized protocol of examinations pre (radiograph, low-dose CT, clinical exam) and postoperatively (radiograph, low-dose CT) before discharge and 6 weeks after (low-dose CT, clinical exam) [41]. The CT preoperative as well as 6 weeks postoperative are part of the clinical standard. Only the CT directly postoperatively takes place in a study-specific manner using the same protocol. 

In both groups, there is a segmentation of the preoperative CT with an analysis of the dysplasia and planning of the correction by CAOS following a standardized protocol. In the CAOS group, 3D-printed patient-specific guides (SLS printed with medical-grade PA12) according to preoperative planning were used to control for the correction amount, while in the control group, conventional methods were used. The postoperative low-dose CT of both groups directly after their surgical correction are compared in terms of improving the acetabular deficit using the 3-DAI as a primary outcome parameter and AV, such as the 3D-HCI, as further parameters. The preoperative 3D-HCI of both groups was compared with the preoperative MI and the AI. With the final low-dose CT 6 weeks postoperatively, consolidation and any loss of correction were assessed. 

### 2.4. Study Intervention

**Figure 4:**
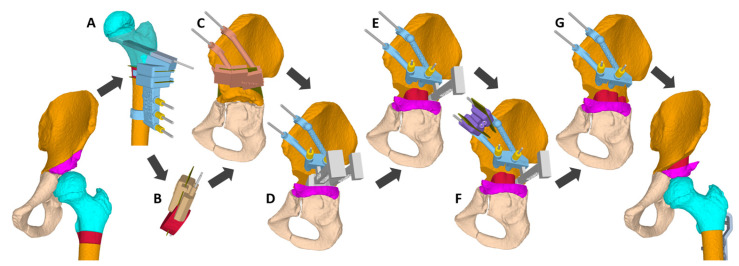
Illustration of hip reconstruction using CAOS. 3D-printed patient-specific guides are used for reorienting subtrochanteric femoral osteotomy (A), cutting the obtained bone block (B), performing the acetabuloplasty (C), maintaining the desired correction (D,E), and completing with an additional bone block harvested from the iliac crest (F), which is placed behind the first block (G).

Both hips of the patients are treated in one surgical session. The same procedure is performed for every patient. Depending on randomization, one side is operated on using conventional methods and the other side according to patient-specific guidelines (CAOS). Using the conventional method, a standardized subtrochanteric osteotomy is performed for varisation and derotation of the femoral head with a shortening of the femur by 10 to 15 mm. The fixation is performed with a Locking Cannulated Blade (LCB) Plate (OrthoPediatrics Corp., Warsaw, IN, USA). The femoral osteotomy is followed by acetabuloplasty. The iliac bone is exposed for about 5 cm from the anterior superior iliac spine along the iliac crest by splitting the pelvic apophysis. The abductor muscles are bluntly detached from the ilium. Now follows the acetabuloplasty to decrease acetabular dysplasia and to enhance containment of the femoral head [41,42]. The osteotomy is performed with a chisel 5 to 10 mm above the acetabular roof in the direction of the triradiate cartilage under fluoroscopic control. Now, the acetabulum can be hinged down to enhance the coverage of the femoral head depending on the deficit of the acetabular roof laterally, ventrally, or posteriorly. In accordance with the gap thus created, there is a suitable bone wedge, which could be obtained by shortening the femur (Figure 4). If necessary, the wedge can be fixed with Kirschner wires. The correction is based on the subjective impression of the surgeon.

Using the CAOS planning of the desired correction 3D-printed patient-specific guides presents the position and direction to perform the femoral and acetabular osteotomy and defines the plate position. A reduction guide defines the acetabular correction and positioning of the bone block to be inserted. The shape of the block is previously adjusted by an additional guide. An additional guide is used to harvest an extra bone block from the iliac crest, which is placed dorsal to the first block.

### 2.5. Eligibility Criteria

In a randomized controlled trial, all children with bilateral CP scheduled for a bilateral hip reconstruction surgery were included. The recruitment and information on both possible surgical methods of the study was conducted after the decision for hip reconstruction. The final consent to participate in the study was given in the following consultation by signature, which always takes place in the clinical routine, so as to carry out the CT to plan the operation and to clarify any remaining questions.

Inclusion criteria:

– Patients with bilateral cerebral palsy, GMFCS levels I to V.
– Minimum age of 4 years and open triradiate cartilage.
– MI ≥ 40% of at least one hip.
– Indication for surgery on both hips.
– CT with possible 3D reconstruction.
– Signed ethical consent.

Exclusion criteria:

– MI ≥ 100%.
– Relevant lumbar scoliosis (>20°) associated with pelvic obliquity.
– Limited compliance for examinations.
– Claustrophobia in CT.
– Withdrawal of study.

The parents/legal guardians and patients were informed about the study. The parents/legal guardians signed the consent form for their children, as they were not able to sign due to their medical situation. If the patient was aged 14 or older and able to assess the situation for themselves and sign, they signed themselves. If the patient was capable of consent, the patient was asked if they wanted to participate in the study. This could be indicated by an affirmative or nodding. If it was obvious that a patient refuses to participate, e.g., by behavioral indications of dissent, the patient was not included.

If patients needed to be excluded after inclusion in the study, additional patients were recruited so the case number was reached.

Drop-out criterion for the patients represents withdrawal of consent.

### 2.6. Power Analysis

We used the superolateral index published by Chung et al. as the primary outcome parameter for assessing the acetabular situation [25,37]. The calculation of the number of cases is based on a project carried out by Chung et al., which uses the same surgical procedure as in the currently planned study (control group). The mean of the postoperative outcome of Chung’s project was at 18° (5°) [37]. In our project, it was expected that the outcome of the group operated on with CAOS would show a clinically relevant difference in the mean of 3.5°. The significance level is 0.05 with a power of 0.80. The calculation results in 32 hips per group. With a drop-out of 20%, this equates to 39 hips per group.

### 2.7. Statistical Methods

The data of the participants were analyzed in the group to which they were randomized (per protocol). The control group was formed by each patient (the second leg) for themselves as a within-patient comparison. If patients dropped out of the study early, the analysis followed an intention to treat approach. All analysis took place after all planned participants had been included and completed follow-up. No stratified analyses were performed.

The radiological variables like MI and AI were measured in the X-ray (preoperatively and directly after surgery), the CT (preoperatively, directly after surgery, and 6 weeks postoperatively), and the 3D segmented reconstructions. In addition, the 3-DAI and 3D-HCI values were determined in each case. The clinical examination (preoperatively and 6 weeks postoperatively) included passive joint motion, muscle strength, and muscle tone of severe joints and muscles on the lower extremities. Furthermore, operation-related variables such as the duration of the operation separately for the pelvis and femur, the length of the scar on the pelvis and femur, blood loss, the estimated extent of correction, and any adverse effects were noted during the operation. These data can be ordinal or scaled.

Descriptive statistics are used to display the results. The radiological variables measured by X-ray or 2D or 3D measurements were used to compare the differences between both groups at every time point. To analyze the alterations between two measurements within every group, differences of radiological variables were calculated (postoperative value, preoperative value). All variables were first tested for normal distribution. For metric tests, mean and standard deviation are reported, and for non-metric tests, median, minimum, and maximum are reported. Both hips were analyzed. To account for bias—the dependence between both legs of a person—the linear mixed model was used for the comparisons. The significance level is 0.05. To analyze the influence of operation-related variables (e.g., the duration of the surgery, or blood loss) and the severity of the preop hip morphology measured by the radiological aspect or the clinical examination, Pearson’s correlation coefficient was used for normally distributed data and Spearman’s rank coefficient was used for non-normally distributed data. Statistical methods were chosen depending on the type of data and the normal distribution. Further subgroup analyses are not planned so far.

The project is supported by a biostatistician over the entire duration as required. SPSS (version 28.0.0) is used for statistical analysis.

### 2.8. Randomization

Patients affected bilaterally are included in the project. Each side of the patient is assigned to one of the two surgical techniques (CAOS or conventional group) by means of block randomization. Block randomization determines whether the more or less affected side is assigned to a technique. This ensures that the more severely affected sides are evenly distributed between the two groups. The severity of the condition is measured by measuring the MI in the preoperative X-ray.

### 2.9. Blinding

The randomization and thus also the blinding of the patients described below took place after the CAOS planning was completed. Patients and parents/legal guardians were blinded to the planned surgical method for the whole treatment. This insured an unbiased assessment of the subjective outcome. The surgeon, the medical doctor hired for the project, and the study nurse who initiated the printing of the guides were unblinded. This is necessary for the further planning of the operation and its implementation. The other people involved in this study, such as the researcher, engineer, and the CAOS group, remained blinded until the decision of which hip would be reconstructed with CAOS.

### 2.10. Outcome

Primary outcome

Research question 1: For the primary research question, the 3-DAI was used as a primary parameter [25]. The purpose of the primary research part is to determine in which intervention group there is a greater improvement in the acetabular coverage. To express this improvement, the difference of the 3-DAI before surgery to 3-DAI at 6 weeks after surgery was calculated separately in both groups. The differences between both groups were compared.

The actual correction was represented by the difference of 3-DAI before surgery to 3-DAI immediately after surgery. To analyze the correction accuracy, this difference of the CAOS group was compared to that of the control group.

Furthermore, the loss of correction was indicated by the difference of 3-DAI immediately after surgery and 3-DAI at 6 weeks after surgery. The differences between both groups were compared to detect a possible loss of correction in one method.

In addition, it was checked which group (CAOS or control group) had a more accurate correction. For this purpose, the 3-DAI of the CP group at 6 weeks after surgery was compared to the 3-DAI calculated as the correction target in the CAOS planning.

Secondary outcomes

Research question 4: To investigate the comparability of the 3D-HCI and 3-DAI, the differences between both variables before surgery and 6 weeks after surgery were calculated. The difference of 3-DAI was compared with the difference of 3D-HCI. These calculations and comparisons were performed separately in the CP group and typically developing reference group.

Research question 5: To identify whether the 3D-HCI is more sensitive than the 3-DAI, data from the typically developing reference group from the retrospective part of the study and from the CP group at 6 weeks after surgery from the prospective study were used. The 3D-HCI of the CP group 6 weeks after surgery was compared with that of the typically developing reference group. The same comparison was also performed with the 3-DAI.

Furthermore, the difference between the 3D-HCI and the 3-DAI of the reference group to those of the CP group was calculated. The difference of the 3D-HCI was compared with that of the 3-DAI to work out differences of the variances of the variables.

## 3. Ethics and Dissemination

This study aims to find out whether femoral head coverage can be performed more precisely by means of a specific surgical procedure. The results will serve in the later application, in case of a better procedure to apply this in the future.

Since to our knowledge, both methods provide good results, there is no increased risk. Since both surgical procedures produce an improvement of the bony situation and for a better comparability of the two procedures, both methods will each be performed on one leg of the same patient. The participating patients received surgery in a clinical routine to avoid major physical damage later, to improve their life situation (e.g., mobility and pain reduction), and to maintain the best possible quality in the long term.

Since the problem already occurs in children and adolescents and the surgery must be performed before the growth plate closes, children and adolescents were included in this study. Patients participated voluntarily and could withdraw consent at any time without giving reasons. This did not affect their medical treatment.

As the patients were under medical treatment, incidental findings will be discussed with them.

### 3.1. Risk-Benefit Assessment

Since the surgical procedures are used regularly, there was no risk for the patients during this study. During the operation, it was possible to switch from the 3D method to the conventional method at any time if the progress of the operation was unacceptable. Through additional evaluations, the results can be viewed more accurately and, if necessary, responded to individually in the treatment context.

The CT before surgery is study-specific and was used to determine the immediate postoperative result. Furthermore, it was used for comparison to 3D planning and for the correction loss of the immediate postoperative result. Everything else took place in the clinical routine. The additional study data are stored in coded form.

### 3.2. Risk of Bias

This study was designed as a randomized within-patient controlled trial. By assigning one hip to the CAOS intervention and the contralateral hip to the conventional procedure, inter-individual confounding factors such as age, GMFCS level, and overall disease severity are inherently controlled.

Selection bias is minimized through block randomization based on the preoperative migration index to ensure balanced allocation of more and less affected hips.

Blinding of the surgeon was not feasible due to the nature of the intervention. However, both hips are operated during the same surgical session by the same surgeon, minimizing systematic differences in perioperative conditions. Two experienced surgeons alternated between cases to reduce potential learning curve effects.

Detection bias was limited as the primary outcome is based on standardized quantitative CT measurements. Statistical analyses account for the non-independence of hips within patients using linear mixed models.

## 4. Discussion

There are, to the best knowledge of the study group, no other projects that address this relevant issue accordingly. However, since the precision of surgical correction for the outcome of the surgery is essential, there is a major need to further investigate such methods to improve the intraoperative control for correction. This is the first study that uses CAOS technology to objectify the lack of acetabular coverage in CP, plan and perform patient-specific correction osteotomies on the acetabular roof, and compare the results with conventional techniques known to show great variability. The results of this study are crucial for the anatomically correct treatment of dysplastic acetabular coverage in CP. Furthermore, this study provides the basis for a better three-dimensional anatomical understanding of dysplasia in this patient group and brings the results with a new 3D-HCI into correlation with the 3-DAI, the AV, and the previously used MI and AI as predictive values as the basis for the indication of surgery.

A disadvantage, besides the additional costs and the time required for planning, is the general accessibility of many centers to CAOS Technology.

Developing and continuously optimizing the guides presents a challenge. It is crucial to accommodate the soft tissue structure to avoid enlarged surgical access while simultaneously ensuring that soft tissue tension does not compromise the precise positioning of the guides. Added to this are the small anatomical dimensions of the typically young children.

## 5. Conclusions

By directly comparing both surgical techniques within the same patients, this study seeks to determine whether patient-specific 3D planning leads to more precise anatomical correction of acetabular coverage compared to conventional freehand osteotomy. Improved precision may have meaningful clinical implications for long-term joint congruency, load distribution, pain, and mobility outcomes. With the 3D-HCI, we can offer a more precise three-dimensional description of the hip subluxation and acetabular coverage than is possible with two-dimensional radiographs. 

## Data Availability

Data are contained within the article.

## Abbreviations

The following abbreviations are used in this manuscript:

FAV	Femoral anteversion
CCD	Caput collum diaphyseal angle
CP	Cerebral palsy
GMFCS	Gross Motor Function Classification System
MI	Migration index
AI	Acetabular index
CT	Computed tomography
3D-CT	Three-dimensional reconstruction
2D-CT	Two-dimensional reconstruction
3-DAI	Three-directional acetabular index
AV	Acetabular volume
MRI	Magnetic resonance imaging
3D-MRI	Three-dimensional reconstruction
CAOS	Computer-assisted orthopedic surgery
HAS	Head-shaft angle
CEA	Center-edge angle
3D-HCI	Three-dimensional head coverage index
